# How Risk Profiles of Investors Affect Robo-Advised Portfolios

**DOI:** 10.3389/frai.2020.00060

**Published:** 2020-09-18

**Authors:** Dmitri Boreiko, Francesca Massarotti

**Affiliations:** ^1^Faculty of Economics and Management, Free University of Bozen-Bolzano, Bolzano, Italy; ^2^Frankfurt School of Finance and Management, Frankfurt, Germany

**Keywords:** FinTech, robo-advisors, investment advise, portfolio management, portfolio optimisation

## Abstract

Automated financial advising (robo-advising) has become an established practice in wealth management, yet very few studies have looked at the cross-section of the robo-advisors and the factors explaining the persistent variability in their portfolio allocation recommendations. Using a sample of 53 advising platforms from the US and Germany, we show that the underlying algorithms manage to identify different risk profiles, although substantial variability is evident even within the same investor types' groups. The robo-advisor expertise in a particular asset class seems to play a significant role, as does the geographical location, while the breadth of the offered investment choice (number of portfolios) across the robo-advisors under study does not seem to have an effect.

## Introduction

Over the last decade, the financial industry experienced some radical changes. Following the financial crisis of 2007, increased regulatory burdens on incumbents and wide adaptations of new technologies led to the emergence of a new structure, where some of the areas are dominated by smaller, more efficient start-ups that use internet, blockchain, and social media to create new products for consumers. The financial technologies (FinTech) adaptation is a key strategic advantage on its way to being successful (Jung et al., 2018)^1^. Technological transformation is particularly evident when it comes to wealth management, retail banking, payments, and lending (Metha et al., 2019). The wealth management industry has not only undergone a transformation driven by technology, but there has been a change also in terms of demand that caused the overall increase of assets under management and the emergence of new players. As Blackrock's (2015) highlighted, the demand for financial advice has increased along with the household's level of cash, people's increased longevity, income gaps caused by retirement, and a general lack of financial literacy.

It is argued that Artificial Intelligence (AI) is one of the most promising technologies that would advance the transformation of the finance industry (Park et al., 2016). One of the most disruptive AI applications in finance has so far been the introduction of automated investment managers or digital advisors, more commonly known as robo-advisors (RAs). Based on each investor's characteristics, RAs deliver and execute portfolio allocation advice through automated algorithms on digital platforms. Purportedly, such a service is free from individual human adviser's biases but come at the cost of a one-size-fits-all problem and limitations introduced by the robo-advising algorithm (D'Accunto et al., 2019).

As of now, consumer adaptation to robo-advising services has been rather slow. Several factors are responsible for such dynamics. First is the lower familiarity with AI and robotic technologies of the investing clients who might dislike entrusting their funds to non-human control (Reuba, 2017; Belanche et al., 2019). Several other aspects behind consumer trust, such as service security, information quality, and general proficiency in Internet usage, also play a role in slower adaptation to RAs (Lee et al., 2018).

Second is the problem of supply, induced by the apprehension of investment-advice providers that the robo-advising services would cannibalize higher-margin human investment advice offered by the same firm^2^. In addition, such an online-based service is subject to the problems of consumer loyalty (Luo and Ye, 2019) that might lead to underinvestment from incumbents given the low switching costs. This problem is aggravated by industry rigidity in adopting advanced marketing methods such as, [for e.g., word-of-mouth marketing, social media, and the internet in general (Casaló et al., 2008)]. The final reason is the legal uncertainty that still surround the RAs business, starting from fundamental issues such as whether RAs are subject to “investment advice” regulations (MiFiD2 in Europe) or possible liability risks, currently of great interest as the markets are being hit hard by the COVID-19-induced crisis (Maume, 2019).

The objective of this research is to empirically examine portfolio recommendations from a diversified set of RAs. So far, academic research has mainly focused on traditional human financial advising, their advice variation, biases, and conflicts of interest. Considering the rapid growth in assets under management (AUM) over recent years^3^, we believe it is necessary to empirically investigate portfolio recommendations provided by automated investment managers.

Previous research has highlighted the variability of the recommended asset allocation from different RAs, however, very few (to our knowledge) addressed the questions as to why this is the case and what, in general, affects RAs' portfolio recommendations. In this study we attempt to identify the factors behind the proposed split between asset classes. Following the research conducted by Mankowitz and Skilje (2018) and focusing on RAs offering advice to retail customers in the German and North American markets, we have investigated whether investors' risk profiles, the number of model portfolios offered by each RA, economies of scale, and RAs' target market have any influence on the final proposed asset allocation.

Based on the sample of active RAs operating in the United States and Germany in 2019 and three constructed generic investors' profiles, we obtained the proposed portfolio splits between equity and debt instruments for each combination of investor-type and RAs under study. Further econometric analysis identified significant variations in recommended equity exposure, thus confirming findings by Cerulli Associates (2015). Our results indicate that cross-firm variations are notably evident for the moderate and especially for the conservative investor types; aggressive investor profiles, on the contrary, seem to receive more uniform asset allocation proposals.

Our multivariate analysis used several plausible explanatory factors for the RAs recommendations. It emerged that the most significant factor impacting portfolio recommendation is the risk-profile of the investors, implying that the RAs included in the sample are able to identify their investors' preferences based on the data entered by the client. In addition, economies of scale have proven to be statistically significant, with equity-specialized RAs favoring equity-biased allocations and vice versa, confirming the findings of Baker and Dellaert (2018). Furthermore, in line with previous research conducted by Rieger et al. (2010), the country of origin seems to have a strong effect. Indeed, U.S.-based RAs tend to skew their recommendations toward equity, thus, probably, addressing the more risk-taking investment mentality of U.S. investors. Lastly, the number of model portfolios surprisingly does not influence portfolio recommendations and is not statistically significant under all specifications.

Recently, the robo-advising industry seemed to have lost its momentum. An absence of trust, legal uncertainty, and low profitability impacted on the rates of growth and a more wide-spread adoption of the technology. For example, ABN AMRO shuttered down its RA Prospery because of low profitability compared to the traditional private banking division^4^. However, the recent Covid-19 crisis has led to a higher participation rate and trading activities of the retail investors who have been gambling on the stock market since March 2020 (Economist, 2020; Financial Times, 2020). The imminent cost-cutting programs would increase the interest on the supply side in wider use of RAs in investment advice. Our results show that, although RAs seem to take into account the risk-profiles of investors, there is still large variability in the investment recommendations even for the same risk-type model investor or models produced by RAs in different jurisdictions. We call for the faster development of industry standards to instill more trust in consumers. Whether these would be adopted as a code of good practice within the financial industry or imposed by the legislators remains an open question.

The rest of the paper is organized as follows. In Robo-advising process and literature review, we briefly describe the robo-advising process and review accumulated academic literature. In Hypotheses, we formulate our research hypotheses. Methods presents the data set and the econometric methods used. Results discusses the main results. The last section addresses the limitations of the study, outlines potential future research agenda, and concludes the paper.

## Robo-Advising Process and Literature Review

It is argued that robotics, artificial intelligence, and blockchain are currently contributing to the transformation of many aspects of the financial industry (Bayon, 2018). Cocca (2016) identifies two streams of innovation in wealth management: virtualization of the interactions with the substitution of the traditional face-to-face meetings with digital channels, and virtualization of the advisory content. The latter process is exactly what automated financial advisors or RAs offer. By leveraging the mistrust in traditional wealth management companies caused by the financial crisis, RAs are offering alternative ways to invest, purportedly free from the deficiencies of a more traditional approach.

Researchers and regulators have still not given an official definition of robo-advising. As argued by Deloitte (2016), the term “robo” indicates the reduced presence or complete absence of human interaction, with automated mathematical algorithms used to produce customized asset allocations. The term “advising” is used to talk about somebody (in this setting, something) giving advice on a matter such as wealth management. Put together, these two terms refer to online-based portfolio management solutions, tailored mainly to retail investors, and attempting to automate all advisory process stages.

The pioneers of robo-advisory platforms were the US-based firms Betterment and Wealthfront, that began offering investment advice to retail investors in 2010. The American market continues to be the largest and most profitable one. Statista (2019) estimates the assets under management (AuM) in Northern America to amount to circa $740 billion, whereas Central and Western Europe AuM is only $26 billion.

Several factors have facilitated the international proliferation of RAs, such as increased investors' protection regulation, higher usage of smartphones and internet access, and increasing awareness and sophistication of retail investors (Haffenden and Melone, 2016). Yet, despite RAs' robust industry growth rates, many still question the viability of the model. As Morningstar (2018) reports, it costs circa £300 to get a new advised client for a robo-advising business, which then generates only £70 in annual revenue.

When the first RAs emerged in the US in 2010, they represented rather basic online interfaces used by financial managers to control their clients' assets. Further evolution underwent four stages, as described by Deloitte (2016). The first stage envisaged the RAs issuing recommendations based on the results of an online questionnaire filled in by investors. Trades were conducted by investors on a different platform, without banks or brokers supporting the robo-advising process and executing orders. In addition, RAs also issued recommendations on individual stocks and bonds.

RAs 2.0 executed investors' trades in addition to providing them with portfolio recommendations. However, it is still not possible to talk about automated investment managers as there is still a human component; indeed, an investment manager is responsible for the supervision of the investment algorithm and oversees setting the investment rules. RAs 3.0 are currently a mainstream in the market with 80% of active players executing investment decisions and portfolio rebalancing automatically via the algorithms. Fund managers only oversee the whole process with limited human intervention. RAs 4.0 employ self-learning artificial intelligence tools for investment algorithms, with automatic rebalancing between asset classes in reaction to market movements and conditions, always complying with investors' preferences expressed via the questionnaire.

Given the different attitudes of investors toward digitalization, robo-advising can be segmented into two main sectors. The first one is pure robo-advising, which is completely free from human intervention in the advisory process. This results in considerably lower fees compared to traditional advisory services, attracting lower-income clientele. As reported by Ringe and Ruof (2018), pure RAs charged fees ranging between 0.4% (US market) and 0.8% (European markets), compared to human financial advising costing circa 1–2%. Pure RAs have become quite popular due to their propensity to avoid conflict of interests due to automation. Fisch et al. (2017) highlight that RAs are less exposed to conflict of interests due to their higher independence, smaller bias to recommend actively managed funds that generate commissions as a potential additional expense, more transparent cost structures, lower minimum investment requirements, and 24/7 availability.

Once the risk profile has been identified, the RAs usually employ modern portfolio theory to construct an optimal mean-variance allocation (Markowitz, 1952). Several optimization algorithms were tried that would work better for an automated advice design (Chen et al., 2019). The investment assets chosen are usually exchange-traded funds, that allow for passive cheap and liquid indexing strategies when investing in different asset classes. Moreover, continuous rebalancing, monitoring, and 24/7 accessibility can also be automatized (Sironi, 2016; Jung et al., 2019).

However, despite the continuous improvement of RAs and the substantial growth in AuM, the value of assets switching to automated investment managers from human financial advisors remains relatively low (Fisch et al., 2017). As was argued by Faloon and Scherer (2017), the modern RAs' questionnaires fail to uncover individual risk aversion and thus are not suited to model the clients' investment problems. Indeed, there is a tendency to retreat from robo-advising (Murray-West, 2018). A survey conducted by IW Capital (2018) has reported that 38% of investors would not count on digital advisers for managing their assets; many investors have discarded automated solutions because of increased market volatility following some economic events, such as Brexit. Several solutions aimed to alleviate these problems were offered that focused on the types of interactions between the algorithms and consumers (Glaser et al., 2019) or suggestions to demonstrate a higher perceived level of automation (Ruhr et al., 2019). However, the industry response was a move backward to a standard investment model, where investment managers utilize digital services for portfolio-rebalancing or asset allocation to optimize their quality of advisory services within a shorter time. Such a model was termed “hybrid robo-advising.”

Surprisingly, the RAs phenomenon has received more attention in psychological and information-technologies scientific literature than in finance and economics research. D'Accunto et al. (2019) is a noticeable exception. In their study, the authors show that RAs help investors to diversify their portfolios and help to mitigate a set of well-known and frequent behavioral biases.

## Hypotheses

Little academic research has addressed the question of the factors that cause advice variability across Ras, which has been well-documented in industry reports. Cerulli Associates (2015) analyzed the proposed asset allocations made by seven different RAs for a 27-years-old investor, whose investment goal was saving for retirement; the proportion of recommended equity obtained by them displayed substantial variation across the RAs under study, with recommendations ranging between 51 and 90%. The recommended exposure to fixed income also seemed to be non-uniform, fluctuating from 10 to 40%.

Foerster et al. (2015) have conducted a study on traditional financial advisors: using regression analysis, they have been able to demonstrate that some advisors, employed at traditional financial firms, fail at tailoring portfolio recommendations to their clients' individual needs and financial situations. Indeed, they have demonstrated that personal characteristics, such as the risk profile, only explain 12.2% of cross-firm variations in recommended equity exposure. According to Lam (2016), this issue can be explained by the fact that portfolio recommendation of traditional financial advisors is driven by their own beliefs, thus implying that they might impose their own opinions on clients' preferences. In contrast to human advisers, RAs tend to provide their recommendations systematically and respect the inputs of the clients; this implies that the proposed asset allocation should be highly influenced by the investors' observable characteristics, in particular by their risk profile. Following the results found by Mankowitz and Skilje (2018), the key factor explaining different weights in asset allocation was found to be investors' risk profiles. According to the authors, digital advisors were able to categorize investors based on their risk-tolerance and they were likely to give them different portfolio recommendations. This might stem from the requirement of financial regulators to provide investors with an asset allocation suitable to them^5^. However, the hypothesis sustained by Lam (2016) and Mankowitz and Skilje (2018) is in contrast with other research and opinions on Ras; automated investment managers have in fact been criticized for their methodology, often considered simplistic and inefficient (Tertilt and Scholz, 2017). Many believe RAs' questionnaires are not as detailed as the ones filled in by human financial advisors. In order to test whether RAs fail to assess their clients' risk profiles, we have formulated our first hypothesis.

*H1: Variations in portfolio recommendation across RAs are explained by their ability to successfully identify investors' different risk profiles*.

A plausible explanation for cross-variation in portfolio recommendation could be the variability in questionnaires' structure and format. One of the more general and frequent differences is the number of questions in the questionnaire. However, previous research (Tertilt and Scholz, 2017) found that it could not explain the investment advice variability. We instead decide to focus our analysis on the RAs' predominance to allocate investors into certain risk categories depending on their answers to the questionnaires. These risk-categories are associated with a certain number of model portfolios, which will be recommended once questionnaires have been answered. Thus, hypothesis two is formulated as follows:

*H2: The ability of the RAs to fully reflect the investor's risk profiles in portfolio recommendation depends on the number of model portfolios offered. Differences in the number of model portfolios offered lead to higher variations in portfolio recommendations*.

As has been mentioned before, RAs are very price-competitive–this is due to economies of scale as the variable costs per additional client are relatively unimportant. Bayon (2018) stresses that RAs also exploit economies of scale as clients' assets are managed based on a limited number of financial products. Baker and Dellaert (2018) put forward a hypothesis that RAs are not more transparent and honest than human financial advisors, and that digital advisors might be programmed to recommend products with the highest margins to the sponsor institution. As a result, it seems that RAs' developers could produce recommendations that would be skewed toward asset classes in which they have higher expertise. Thus, our third hypothesis is as follows:

*H3: Cross-firm variations in portfolio recommendation could be explained by the RAs sponsors' expertise in different asset classes*.

Rieger et al. (2010) have demonstrated that risk behavior varies across countries and cultural regions. Results showed that American investors tolerate more risk than European investors do. We have selected our sample of RAs advising US and German residents, hoping to see some considerable differences across the two regions. Our last hypothesis therefore is as follows:

*H4: German RAs tend to recommend more conservative allocations than their United-States-based competitors, as manifested by the suggested proportion of investment in fixed income products*.

## Methods

### Sample

The focus of this research is on testing what causes variations in portfolio recommendations between RAs based in the United States and in Germany. Our choice of countries was motivated by interest to compare US-based RAs against non-US-based ones, and Germany featured the highest number of active players^6^. The first key and challenging step was in identifying the relevant market players. In the absence of any coherent database of operational RAs, we have relied on market reports (CBInsights, 2017; Fintechnews Switzerland, 2018) and on various reviews and comparisons of RAs found on dedicated blogs (Robo-Advisor Comparison for the United States and ExtraETF for Germany)^7^. From these sources we have constructed an initial sample of 84 active digital advisors, based either in Germany or in the United States. We excluded RAs that were based in other countries but offered the services to US and German citizens, B2B advisors, or the ones that serviced a restricted group of investors^8^. The final sample consists of 62 B2C-oriented RAs. We also had to exclude some services that required a social security number as an input, leaving 53 RAs in the final sample. Of the RAs, 28 are based in Germany, while 25 of them are in the United States.

The pure RA model is prevalent in our sample–only 19 RAs offer the possibility to talk to a human financial advisor at some stage. This is in line with the increasing tendency to switch to hybrid robo-advisory, as more established financial institutions are launching their own robo-advising platforms, such as Charles Schwab in the United States and Castell'sche Bank in Germany.

We controlled the fees charged by RAs in our sample across the industry reported figures. As it can be seen in Table 1, U.S.-based pure-robo advisors included in the dataset show an average of 0.34% management fee, while German-based players tend to have higher fees of circa 0.9%. Hybrid RAs in our sample tend to be more expensive than pure RAs, regardless of the country of origin. Both findings are in line with industry-reported numbers.

**Table 1:** Average management fees charged.

	**United States** ***N* = 25 %**	**Germany** ***N* = 28 %**	**Diff. in means** **(Germany vs. US) %**
Pure robo-advisors (*N* = 34)	0.34	0.90	+0.56^***^
Hybrid robo advisors (*N* = 19)	0.36	1.06	+0.80^***^
All (*N* = 53)	0.35	0.95	+0.60^***^

*The Table reports the level of management fees by country and by RA types. Pure/Hybrid RAs stand for services offered without / with possibility of human adviser interaction*.

*** *denote the significance of the respective coefficients at 1% level*.

To help investors to make conscious and profitable investment decisions, it is essential for an advisor to successfully identify their risk tolerance; the failure to do so might lead to the selection of sub-optimal asset allocation. For the sake of comparing portfolio recommendations, we have created the following three general investor profiles with varying risk attitudes that we called “conservative,” “moderate,” and “aggressive” investor's types. The assembled investors' profiles were fed to online questionnaires from 53 RAs, resulting in a collection of 159 portfolio recommendations^9^. The detailed information about the risk profiles' construction is given in Appendix 1. Table 2 provides an overview of the general investors' profiles.

**Table 2:** Investors' profile descriptions.

	**Conservative**	**Moderate**	**Aggressive**
Age	48 years old	48 years old	48 years old
Gender	Male	Male	Male
Education	High school diploma	High school diploma	High school diploma
Marital status	Married	Married	Single
Dependents	Yes	No	No
Field of work	Logistic	Logistic	Logistic
Annual income	$63,875^a^ (€57,000)	$63,875 (€57,000)	$63,875 (€57,000)
Aim of investment	Saving for retirement	Saving for retirement	Saving for retirement
Investment philosophy	Minimize losses	Minimize losses and maximize returns	Maximize returns
Investment horizon	3–5 years	3–5 years	3–5 years
Risk tolerance	Low	Medium	High
Amount invested	$6,386	$6,386	$6,386
per year	€5,700	€5,700	€5,700

a *EUR/USD exchange rate as per 14.05.2019*.

The recommended ratio of the investment in equity class was taken as the dependent variable. It was calculated in the following way:

(1) $$\begin{array}{r} y_{ij}=\;\frac{{\;RE}_{ij}}{RE_{ij}+RFI_{ij}+ROtA_{ij}} \end{array}$$

$$\begin{array}{l} i\varepsilon \{1,\;2\ldots .53\}\\ j\varepsilon \{conservative,\;Moderate,\;Agrressive\} \end{array}$$

In Equation 1, *y*_*ij*_ is the ratio of the recommended equity ( *RE*_*ij*_) in the recommended portfolio composed of equity, fixed income, and other assets (*RE*_*ij*_ + *RFI*_*ij*_ + *ROtA*_*ij*_).

### Estimation Methods

We used the ordinary least square (OLS) regression model with corrections for autocorrelation and heteroscedasticity based on the Newey–West method. Considering the full sample and the formulated hypothesis, the following model has been tested:

(2) $$\begin{array}{l} y_{ij}=\;\alpha_{i}+\;\beta_{1}Country_{i}+\beta_{2}Moderate_{i}+\;\beta_{3}Conservative_{i}\\ \;+\;\beta_{4}{Portfolios\;Offered}_{i}+\beta_{5}Equity\;Expertise_{i}\\ \;+\beta_{6}Fixed\;Income\;Expsrtise_{i}\\ \;+\beta_{7}Other\;Asset\;Expertise_{i}+\varepsilon_{i} \end{array}$$

Appendix 2 defines all the variables and their data sources. In order to test for the hypotheses formulated earlier, the independent variables for assessing the number of portfolios offered, the equity, and the fixed income expertise have been introduced. The numbers of portfolio offered, the key variable for testing for H2, is determined by manually counting the model portfolios offered by each RA. This information can be generally found on the website of the digital advisors; in some cases, however, it has been necessary to directly contact the provider for more detailed information. The number was in all cases double-confirmed during the portfolio recommendation phase. The RAs in our sample offer on average seven model portfolios, with German RAs offering 7.7 portfolios, and US ones only 6.2.

A proxy capturing the expertise in investing in equity, fixed income, and other assets of each RAs was created in order to test the third hypothesis. RA's expertise has been proxied with the weights, *w*_*ij*_, of each investment class in the RAs investment universe. These have been calculated as the following:

(3) $$\begin{array}{r} w_{ij}=\;\frac{{\;n}_{ij}}{m_{j}} \end{array}$$

Where, in Equation 3, *n*_*ij*_ indicates the number of the assets within asset class *i* and per RA *j*, while *m*_*j*_ represents the total number of assets in each RA investment universe. Securities were categorized following the industry convention by dividing them into equity, fixed income, and other assets groups. The categorization has been done manually based on the information provided by the digital advisors. In most cases, the list of investment vehicles is publicly available; when this was not the case, RAs were directly contacted.

Table 3 shows that US-based RAs tend to have more exposure to equity than their Germen peers. It is known that the capital markets' participants in the United States tend to be more likely to be risk-takers than their counterparts in Germany, who generally have a more conservative approach. In addition, it could be said that, in both countries, lower importance is given to the other assets; this could be explained by the fact that most of the digital advisers taken into consideration for this study do not include “other assets” in their investment universe.

**Table 3:** Expertise in asset classes vs. geographical location.

**Country**	**Variable**	**Mean**	**Std. Dev**	**Min**	**Max**
United States	Expertise in equity	0.51^**^	0.24	0.01	1.00
	Expertise in FI	0.28	0.18	0.00	0.71
	Expertise in other assets	0.15	0.14	0.00	0.46
Germany	Expertise in equity	0.44^**^	0.21	0.14	1.00
	Expertise in FI	0.36^**^	0.15	0.00	0.60
	Expertise in other assets	0.20	0.14	0.00	0.50

*The Table reports the statistics of expertise in various asset classes by geographical location. Pure/Hybrid RAs stand for services offered without/with possibility of human adviser interaction*.

** *denote the significance of the respective coefficients at 5% level*.

## Results

### Univariate Analysis

In line with results found by Cerulli Associates (2015), even for the same risk profile, significant variations in asset allocation across RAs have been found. This is particularly true for equities and fixed income, characterized by higher standard deviations than the other assets, with the results reported in Table 4. The table displays the descriptive statistics for portfolio recommendation subdivided by asset classes and by above-described general investors' profile. Interestingly, results display that the conservative investors experience higher variances in recommended allocation across all asset classes.

**Table 4:** Descriptive statistics for portfolio recommendations.

**Investor style**	**Advice**	**Mean**	**Std. Dev**	**Min**	**Max**
Aggressive	Equity	0.73^***^	0.23	0.18	1.00
	FI	0.21	0.21	0.00	0.75
	Others	0.06	0.07	0.00	0.33
Moderate	Equity	0.56^**^	0.23	0.14	1.00
	FI	0.38^*^	0.21	0.00	0.75
	Others	0.06	0.08	0.00	0.41
Conservative	Equity	0.35	0.28	0.00	1.00
	FI	0.59^**^	0.27	0.00	1.00
	Others	0.06	0.09	0.00	0.51

*The Table reports average values of recommended allocations to various asset classes by the investor risk profile. FI stands for Fixed Income investment class*.

***, **, * *denote the significance of the respective coefficients at 1,5, and 10% levels*.

### Regression Results

Table 5 reports the results of the OLS regressions. The first regression excludes control variables. As predicted, more risk-averse profiles result in smaller share equity allocation. As seen in regression 2, US-based RAs generally recommend a higher investment in equity, even after controlling for all other factors. The breadth of the portfolio choice does not play a significant role, still RA's equity expertise positively affect allocation to equity asset class.

**Table 5:** Recommended allocation to equity.

	**(1)**	**(2)**	**(3)**	**(4)**
Moderate	−0.174^***^ (0.028)	−0.174^***^ (0.027)	−0.174^***^ (0.028)	−0.167^***^ (0.027)
Conservative	−0.376^***^ (0.042)	−0.376^***^ (0.041)	−0.376^***^ (0.041)	−0.373^***^ (0.039)
Location: USA		0.175^**^ (0.053)	0.179^**^ (0.053)	0.084^*^ (0.043)
N of Portfolios Offered			0.002 (0.005)	0.000 (0.002)
Expertise in Equity				0.367^***^ (0.074)
Expertise in FI				−0.351^***^ (0.104)
Expertise in Other Assets				−0.154 (0.193)
Intercept	0.073^***^ (0.035)	0.647^***^ (0.044)	0.627^***^ (0.053)	0.649^***^ (0.084)
Adj. R^2^	0.28	0.37	0.37	0.58
N obs.	159	159	159	159

*The Table reports the results of the OLS regressions of recommended portfolio allocation to equity (in % to total investment) against the risk profile of the investors and other explanatory variables. All variables are defined in Appendix 2*.

***, **, * *denote the significance of the respective coefficients at 1,5, and 10% levels*.

As can be seen in Table 5, the risk profiles are strongly statistically significant and the intercept, e.g., the *Aggressive*_*i*_ risk profile variable, is positively correlated with the equity investment recommendations. In the case of risk-taking investors, the share of equity in the recommended portfolio is likely to increase by 64.9%. On the contrary, if the investor has a moderate risk-tolerance or is risk-averse, RAs tend to recommend the final portfolio that feature a smaller equity stake (16.7 and 37.3%, respectively). This said, we fail to reject the first hypothesis that variations in portfolio recommendation across RAs are explained by the digital advisers' ability to successfully identify investors' different risk profiles.

We also demonstrate that the choice of portfolios offered does not influence the portfolio recommendation, hence they do not cause cross-firm variations. The variable *N. of Portfolios offered* is insignificant in all the regressions and this provides evidence against the second hypothesis. We find that differences in the number of model portfolios offered across various RAs do not lead to variations in portfolio recommendations.

In line with the economies of scale hypothesis, it has been found that RAs with more expertise in equities tend to base their recommendations more on this particular asset class. The regressions show that for these RAs the recommended share of equity is higher (36.7%). On the contrary, if the RA proves to have more expertise in fixed income, the weight toward equity for the recommended asset allocation is lower (−35.1%). As can be seen from Table 5, both *Expertise in Equity* and *in FI* variables are found to be strongly statistically significant across all regressions. Therefore, we find support for the third hypothesis for equity and fixed income expertise, but not for the *other assets* class.

Lastly, it also emerges that the USA domicile dummy is statistically significant. The US-based RAs generally recommend higher equity allocations (by 8.4% on average). Thus, in line with previous expectations and the related literature, geographical location does play a role in recommended portfolios. Therefore, we also confirm our fourth hypothesis of US-based RAs advice to be skewed to the equity assets.

We also run similar regressions for conservative, moderate, and aggressive investor groups separately. As can be seen in Table 6, the only variable that is statistically significant across all the three profiles is the equity expertise. This implies that the main factor affecting the increase of the equity portion in portfolio recommendation is RAs' experience in investing in equity products, thus confirming the economies-of-scale hypothesis (H3). Moreover, the expertise in fixed income has proven to be strongly statistically significant for both the conservatives and the moderate investors. Some expertise in FI investment reduces the recommended equity exposure by more than 40% for the conservative and moderate investors.

**Table 6:** OLS Regression Results for each investor style.

	**Conservative**	**Moderate**	**Aggressive**
Intercept	−0.011 (0.083)	0.700^***^ (0.065)	0.716^***^ (0.089)
Country	0.037 (0.064)	0.074 (0.052)	0.142^*^ (0.064)
Portfolios offered	0.004 (0.004)	0.000 (0.003)	−0.004 (0.004)
Equity expertise	0.800^***^ (0.08)	0.142^*^ (0.061)	0.156^*^ (0.076)
Fixed income expertise	−0.414^***^ (0.112)	−0.548^***^ (0.102)	−0.090 (0.11)
Other assets expertise	0.349 (0.244)	−0.404 (0.223)	−0.409 (0.240)
R^2^	0.61	0.52	0.33
N obs.	53	53	53

*The Table reports the results of the OLS regressions of recommended portfolio allocation to equity (in % to total investment) for each risk profile subsample against the selected explanatory variable. All variables are defined in Appendix 2*.

*** and

* *denote the significance of the respective coefficients at 1% and 10% levels*.

## Discussion and Conclusions

In this study we have identified some of the factors influencing portfolio recommendations provided by RAs and thus causing cross-firm variations. Using a sample of cross-sectional data containing the asset allocation recommendations provided by 53 different digital advisors based either in the United States or in Germany, we analyzed whether RAs comply with financial regulations and recommend investors with different risk-preferences different portfolios. We find that in our sample, RAs successfully recognize investors' style and provide them with different portfolio recommendations, thus complying with financial regulations. Still, there is large variability in the investment recommendations even for the same risk-type model investor or produced by RAs in different jurisdictions. We call for faster development of the industry standards to instill more trust from consumers. Whether these would be adopted as a code of good practice within the financial industry or imposed by the legislators remains an open question.

We also confirm that the number of portfolios offered is not statistically significant in explaining the recommended equity weight in a portfolio, in line with the results of Mankowitz and Skilje (2018).

Furthermore, the equity ratio is found to have a positive and negative association with RA's equity and fixed income expertise, respectively, providing evidence for the direct effect of the economies of scales. In addition, the study demonstrates the existence of large inconsistencies in portfolio recommendations, especially for moderate and conservative investors. It could be concluded that economies of scale are considered a key factor affecting portfolio recommendation and, it being a firm-specific capability, it can also be considered the main factor causing cross-firm variations.

Lastly, results demonstrate that RAs based in the United States recommend higher equity exposure than German digital advisers. The fact that US-based RAs recommend 8.4% more equity that their German counterparts can be interpreted as a proof of existence of some essential beliefs or investment preferences in the United States that are not shared in Germany.

Certainly, our research design is not without limitations. First of all, our sample might suffer from selection bias since we were not able to include all active RAs in the USA and Germany. However, we believe that we have identified and included in the sample virtually all that are being advertised and that have open access to the general public.

As the study is focused only on the two countries, given the significance of country effect on portfolio recommendation and considering that investors' mentality is highly influenced by their country of origin, other conclusions could be drawn when changing the geographical location or when including other countries in the sample. If, however, an official database with information given by financial regulators would be available to academia, the sample could be enriched, and the generalizability of the study would improve.

Further research on robo-advising industry should focus on more geographically dispersed samples, preferably adding the time-series dimension by looking at the variation of recommendations for each RA across time. Comparing the rates of return earned by investing in recommended portfolios against the human-adviser averages or market indices also merits further attention. It is highly desirable to enrich the set of controls and add more dimensions to the typical risk profiles of investors. The impact of social media certainly deserves special attention. If more information about and from RAs were available, it would be interesting to control for the impact on performance, client attraction, and client retention of new publicity provided by articles published on a dedicated blog such as Extra ETF. However, given the secrecy and confidentiality of these start-ups, it is not possible to capture this effect.

Other potential suggestions for future research would be to focus on the development of the RAs market with the entrance of the established financial service sector actors. However, despite further improvements of the regression model via the enlargement of the sample or a wider choice of explanatory variables, the most challenging aspect of the research on RAs is still the lack of transparency and its effect on trust; considering that RAs are primarily private companies, finding relevant information about their operations, profitability, and business models is quite challenging. To sum up, little is still known about the future of the automated-advice industry and AI applications in finance. The COVID-19 shock to the financial industry is still to be gauged. Whether FinTech will be a victim or a savior remains to be seen.

## Data Availability Statement

The datasets generated for this study are available on request to the corresponding author.

## Author Contributions

DB general structure, literature review, and discussion. FM data collection, empirical analysis, and hypotheses set-up. All authors contributed to the article and approved the submitted version.

## Conflict of Interest

The authors declare that the research was conducted in the absence of any commercial or financial relationships that could be construed as a potential conflict of interest.

## Footnotes

^1^For a brief history of how technological innovations impacted financial industry in the last 75 years, see Ashta and Biot-Paquerot (2018).

^2^This concern was voiced in several interviews with investment management firms we conducted while writing this paper.

^3^S&P (2016).

^4^https://www.finextra.com/newsarticle/33623/abn-amro-shuts-down-german-digital-wealth-manager-prospery (accessed November 08, 2019).

^5^See European Securities Markets Authority (ESMA) (2018). Guidelines on Certain Aspects of The MiFID II Suitability Requirements.

^6^Business Insider Intelligence, “The US still has the robo-advisor lead,” Business Insider. (2017). Available online at: www.businessinsider.de/the-us-still-has-the-robo-advisor-lead-2017-4 (accessed August 20, 2019).

^7^https://www.roboadvisorpros.com/category/comparisons/ and https://de.extraetf.com/robo-advisor

^8^For example, Ellevest, a US-based RA providing advice only for women.

^9^The exact structure of the questionnaires is available from the authors upon request.
