# Supplementary material for: How Risk Profiles of Investors Affect Robo-Advised Portfolios
Source: Front Artif Intell. 2020 Sep 18;3:60. doi: 10.3389/frai.2020.00060 (PMC7861303; doi:10.3389/frai.2020.00060)
Supplement: Supplementary file 1 [file Data_Sheet_1.pdf]

Appendix 1. Risk profiles of the investors

The risk profiles have been constructed based on the minimum requirements set by Article 25 in MiFID II and the Rule 2111 of FINRA for giving any recommendation on financial products. The key profiles' characteristics in our study are as follows.

1. **Age.**

While it is true that the younger generations are generally fonds of technology and that financial-educated millennials might be considered favorable target customers, it cannot be assumed that they are the ultimate users of robo-advisors (Nest Wealth, 2019). Indeed, the average age of robo-advisors' clients is 48, and among those investors 20% is over 62 years old (Kaya, 2017). Several studies of behavioral finance claim that the younger investors are, the higher their risk capacity (Sung & Hanna, 1996). Therefore, it could be argued that fixing a unique age for the three profile might not reflect this behavioral aspect. Further critique could rely on the finding of a study conducted by Giuliano and Spilimbergo (2009) indicating that people experiencing a financial crisis in their 16-25 tend to be more conservative in the future and trust less the stock market. All those aspects combined do not justify the old age given to the aggressive profile. However, it must be pointed out that not only the study is aimed at being general, thus justifying why the average investors' age has been used for the three profiles; but as the U.S. National Institute on Retirement Security confirms, most of the people aged between 21 and 32 years old do not save for retirement, which is the ultimate investment goal of the generic investors. Therefore, setting a younger age for the "conservative", "moderate" or "aggressive" investors would have been in contrast with the purpose of the research.

2. **Gender.**

For the sake of generalization, it has been decided not to differentiate between men and women. Including gender differences in the profiles would require some adjustments in the whole model, indeed it has been demonstrated that women tend to be more risk-averse than men, adding gender discrepancies between the investors' profiles would have added more complexity to the study. Additionally, several studies have reported that women tend to be less involved with the stock market (Barrett, 2016), which led to opting for male general

investors rather than women. Moreover, Lussardi and Mitchell (2008) found out that women with less financial literacy are less likely to save for retirement, which is the goal of the investors considered in this analysis.

3. **Education**.

It has been proved that education plays an important role on the risk profile of investors (Baker and Haslem, 1974; Grable and Lytton, 1998). According to a research conducted by Sulaiman (2012), a higher level of education increases individuals' capabilities of risk assessment and, in general, people with a higher education are found to be more risk tolerant. Nevertheless, we claim exceptions exist and with the additional purpose of reducing complexity, education discrepancies between the three profiles have been avoided. Evidence reported by Anderson and Robinson (2018) shows that overestimation in financial knowledge decreases with the level of education. Along with this, Porto and Xiao (2015) have studied the relationship between financial overconfidence and advice seeking: results have demonstrated that overconfident investors seek advice mainly for debt and tax issues rather than investments. Therefore, it was essential to set an education level that was not too low. Considering the age of the general investors, and the average level of education attained for their generation in both the United States and Germany (United States Census Bureau, 2017; OECD, 2018) a high-school diploma should properly reflect the general education level achieved by the generation they belong to.

4. **Marital status and dependents.**

Previous research confirmed that changes in the two variables might also lead to changes in both the investment mentality and in the aim of investments (Roszkowski et al. 1993). This change might be explained by the increment of responsibilities normally encountered by a married couple. Married couples might also experience increased financial responsibilities in presence of dependents; which in turns leads to the increase of risk-aversion. Hence, the conservative investor is set to be married with children, the investor with moderate risk aversion is married with no children and the more aggressive investor is instead single with no children. Although it could be argued that complexity might be increased, it is necessary to say that the overall picture of the investor is more precise.

5. **Field of work.**

Evidence reported by Chalmer and Reuter (2015) shows that demand of asset allocation recommendation is likely to be higher among those investors with low level of financial sophistication. A lack of financial background is also linked to underestimation/overestimation of total wealth, this generally happens to financial advisors' clients. Additionally, studies conducted by Fisch at el. (2015) demonstrated that the help of a financial advisor is crucial to mitigate the effects of financial illiteracy, in this case the role of the financial advisor will be covered by the robo-advisors. For all these reasons, it has been decided not to link the general investors' profiles to the financial industry.

6. **Income.**

Different figures assessing the average income of RAs' clients have been found; nonetheless, the variance between them is quite high. Hence, the average income by age has been considered depicting the general investors profiles. Giving that the general investor is 48 years old, we have taken the average income for people between 45-54 years old in the US and in Germany, which are the two countries we want to focus on (Nier, 2017). The figures provided seem to be in line with the studies published by Kaya (2017) suggesting that the average income of robo-advisors clients is €54,000 p.a.

7. **Aim of investment.**

Over the last twenty years, public and private retirement plans have moved away from defined benefit schemes switching towards defined contribution schemes (Anderson and Robinson, 2018). As a result, more financial sophistication and in general engagement is needed for taking saving decisions. Giving that many people might not have any financial knowledge they might opt for a robo-advisor for saving for retirement. Hence, this has been set as the aim of investment for the three profiles.

8. **Investment horizon**.

Although the purpose of investment has a long-term focus, it is widely known investors often act irrationally (Shiller, 2016). In order to insert some common investors mistakes, a short-term investment horizon has been set. Scalable Capital reports in its whitepaper that a 1-year horizon has been used for simulations once the clients' VaR has been set, implying that the

decision taken conforms with industry practices. No unique average figure for the average investment horizon has been found: Wealthfront, for example, declared their clients' investment horizon is relatively high without giving any specific number. Betterment uses a 30-years' time horizon for its estimations.

9. **Annual Investment.**

This amount might be low for high-net worth individuals; however, robo-advisors aim at targeting those investors who could not afford a financial advisor. On average, the minimum investment amount necessary to open an account is set at €5,000. Additional evidence reported by Kaya (2017) states that the general robo-advisor client invests between €1,000 and €1,500 p.a. Therefore, in absence of further official information provided by the robo-advisors, an investment amount of 10% of an average salary has been selected.

Appendix 2. Variables definitions.

	Definition	Data source
Dependent variable		
Recommended equity allocation, %	Calculated as the proportion of total investment funds to be allocated to equity class as advised by the RAs in the sample.	RAs portals
Independent variable		
Aggressive / moderate / conservative risk profile dummy	Equals to 1 if the investor is of aggressive, moderate, or conservative risk profile, respectively.	Authors' calculations
Location: USA dummy	Equals to 1 if the RA is domiciled in the United States and 0 otherwise.	RAs portals
Number of portfolios offered	N of model investment portfolios offered by RAs	RAs portals and information offices; web search
Expertise in Equity / FI / Other Assets, %	Proportions of Equity, FI, or Other Assets classes in the investable universe of each RA	RAs portals and authors' calculations
